# SNX10 and PTGDS are associated with the progression and prognosis of cervical squamous cell carcinoma

**DOI:** 10.1186/s12885-021-08212-w

**Published:** 2021-06-11

**Authors:** Pinping Jiang, Ying Cao, Feng Gao, Wei Sun, Jinhui Liu, Ziyan Ma, Manxin Xie, Shilong Fu

**Affiliations:** 1 Department of Gynecology, The First Affiliated Hospital of Nanjing Medical University, 300 Guangzhou Road, Nanjing, 210029 Jiangsu China; 2 Department of Obstetrics and Gynecology, Changzhou Second People’s Hospital, Changzhou, 213000 Jiangsu China; 3 Department of Orthopedics, The First Affiliated Hospital of Nanjing Medical University, Nanjing, 210029 Jiangsu China; 4 University of New South Wales, Sydney, Australia

**Keywords:** Cervical squamous cell carcinoma, Methylation, Prognosis, Risk model, SNX10, PTGDS

## Abstract

**Background:**

Cervical cancer (CC) is the primary cause of death in women. This study sought to investigate the potential mechanism and prognostic genes of CC.

**Methods:**

We downloaded four gene expression profiles from GEO. The RRA method was used to integrate and screen differentially expressed genes (DEGs) between CC and normal samples. Functional analysis was performed by clusterprofiler. We built PPI network by Search Tool for the Retrieval of Interacting Genes Database (STRING) and selected hub modules via Molecular COmplex Detection (MCODE). CMap database was used to find molecules with therapeutic potential for CC. The hub genes were validated in GEO datasets, Gene Expession Profiling Interactive Analysis (GEPIA), immunohistochemistry, Cox regression analysis, TCGA methylation analysis and ONCOMINE were carried out. ROC curve analysis and GSEA were also performed to describe the prognostic significance of hub genes.

**Results:**

Functional analysis revealed that 147 DEGs were significantly enriched in binding, cell proliferation, transcriptional activity and cell cycle regulation. PPI network screened 30 hub genes, with CDK1 having the strongest connectivity with CC. Cmap showed that apigenin, thioguanine and trichostatin A might be used to treat CC(*P* < 0.05). Eight genes (APOD, CXCL8, MMP1, MMP3, PLOD2, PTGDS, SNX10 and SPP1) were screened out through GEPIA. Of them, only PTGDS and SNX10 had not appeared in previous studies about CC. The validation in GEO showed that PTGDS showed low expression while SNX10 presented high expression in tumor tissues. Their expression profiles were consistent with the results in immunohistochemistry. ROC curve analysis indicated that the model had a good diagnostic efficiency (AUC = 0.738). GSEA analysis demonstrated that the two genes were correlated with the chemokine signaling pathway (*P* < 0.05). TCGA methylation analysis showed that patients with lowly-expressed and highly-methylated PTGDS had a worse prognosis than those with highly-expressed and lowly-methylated PTGDS (*p* = 0.037). Cox regression analysis showed that SNX10 and PTGDS were independent prognostic indicators for OS among CC patients (*P* = 0.007 and 0.003).

**Conclusions:**

PTGDS and SNX10 showed abnormal expression and methylation in CC. Both genes might have high prognostic value of CC patients.

**Supplementary Information:**

The online version contains supplementary material available at 10.1186/s12885-021-08212-w.

## Background

An annual death toll of 265,700 makes cervical cancer (CC) the second deadliest malignancy in females [1]. Despite pre-cancerous screening and emerging treatments, CC remains the primary cause of death in women in developing countries [2]. When CC metastasizes and recurs, the prognosis gets even worse. Therefore, it is of great significance to create new treatments of CC based on its to-be-clarified molecular mechanism.

Gene expression microarray, as an efficient means of acquiring large-scale genetic data, is being widely used to study gene expression profiling in many human cancers. Upon microarray and databases, effective analytic tools have been designed to explore tumor-associated genes, molecular mechanisms and target therapies. The integration of databases containing gene expression chips allows in-depth study of molecular mechanisms [3, 4].

Thousands of differentially expressed genes (DEGs) in CC have been discovered [5–7]. However, the results on some mRNAs are inconsistent. Here we use an unbiased approach to solve this problem.

**Fig. 1:**
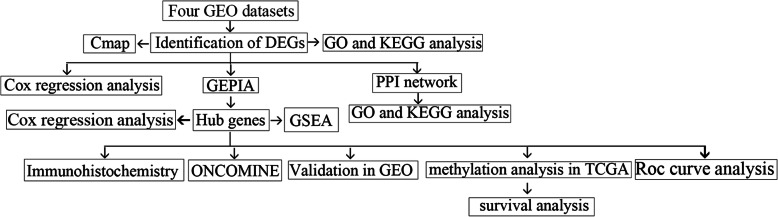
Flow chart of the present study

In our study, we screened DEGs from four profiles downloaded from GEO. PPI network was built by STRING Database and hub modules selected via plug-in MCODE. CMap was used to find potential genes associated with CC. We also validated hub genes with GEO, GEPIA, immunohistochemistry and ONCOMINE. ROC curve analysis and GESA were also done to tease out the significance of hub genes. The flow chart of this research was displayed in Fig. 1.

## Methods

### Screening DEGs

Keywords “cervical cancer geo accession” were put in the GEO database (https://www.ncbi.nlm.nih.gov/geo/) and the mRNA expression profiles of GSE6791, GSE63514, GSE39001 and GSE9750 were downloaded. The dataset details were shown in Table 1. We processed unqualified data by R package. The data is calibrated, standardized and log2-transformed. Gene expression analysis was performed using the “limma” R package [8] in the Bioconductor package. Relevant codes were placed into R. we selected four microarray datasets and analyzed them with limma. The |log2fold change (FC)| > 2 and adjusted *p* < 0.05 were set as cutoffs. RRA package was download (http://cran.r-project.org/) [9] and R software was used for running the instruction code.

**Table 1:** Details for GEO cervical cancer data

Reference	Sample	GEO	Platform	Normal	Tumor
Pyeon D,et al. (2007) [56]	Cervix	GSE6791	GPL570	8	20
Scotto L,et al. (2007) [57]	Cervix	GSE9750	GPL96	24	33
Espinosa AM,et al. (2012) [58]	Cervix	GSE39001	GPL201 GPL6244	12	43
den Boon JA,et al. (2014) [59]	Cervix	GSE63514	GPL570	24	28

### Functional analysis based on DEGs

The biological function of DEGs was analyzed with DAVID (https://david.ncifcrf.gov/) database and clusterprofiler [10] (a package visualizing the biological profiles of genes). *P* < 0.05 was considered to be statistically significant.

### PPI network integration

Search Tool for the Retrieval of Interacting Genes Database (STRING) [11] (http://www.string-db.org/) was used to assess PPI complex between identified and predicted proteins. In addition, the plug-in MCODE [12] of Cytoscape was conducted to select and visualize hub modules in PPI complex.

### Identification of potential drugs

CMap [13] is a computer simulation method for predicting the potential drug that may induce or reverse a biological state encoded by the gene expression signature. The different probe components commonly between CC and normal samples were screened out with CMap database and divided into the up- and down-regulated groups. An enrichment score representing similarity was calculated. The positive score illustrated that the drug could induce cancer in human; the negative score illustrated the drug function oppositely and had potential therapeutic value.

### Construction of a prognostic signature

Univariate Cox regression analysis was performed based on DEGs. The genes associated with prognosis were defined using the cutoff value of *p* < 0.05. Next, a multivariate Cox regression model was constructed with genes of *p* < 0.01. Cox regression with *p* < 0.05 was constructed to estimate the risk score of each patient on the expression of the DEGs. To further screen out prognosis-related genes of CC, we constructed a linear risk model. The prognostic score = expRNA1 × βRNA1 + expRNA2 × βRNA2 + expRNA3 × βRNA3 + ...expRNAn×βRNAn (expRNA is the expression level of each methylation-driven gene, and βRNA is the regression coefficient calculated by the multivariate Cox regression analysis). The prognostic risk value of each sample was calculated according to the formula, and then the median of the index value was cut off. Patients were separated into a low- and high-risk group according to their mean scores of prognostic risks. Kaplan-Meier survival analysis was conducted based on the low- and high-risk group. We also performed ROC curve analysis using 5 years as the predicted time to assess the predictive value of the outcome. The areas under the ROC curve, sensitivity and specificity were used to describe predictive values.

### Validation of key genes

We used GEPIA [14] (Gene Expression Profiling Interactive Analysis) to analyze the expression and prognostic significance of DEGs. After reviewing literature, we screened real hub genes from DEGs. Subsequently the real hub genes were validated into GEO datasets, including GSE7803 and GSE29576, and ONCOMINE database (www.oncomine.org). GSE7803 included 21 cervical cancer samples and 10 normal cervix tissue samples. GSE29576 included 45 CC samples and 17 normal cervix tissue samples. ONCOMINE [15] dataset is a public online cancer microarray database that enables online analysis on relations between certain gene and various tumors according to DNA and RNA sequence data. The Human Protein Atlas (HPA) (http://www.proteinatlas.org/) was also used to measure the expressing level of the real hub genes. ROC curve analysis was performed to distinguish normal and cancer tissues.

### Survival analysis and mapping of methylation level

Survival analysis on gene methylation and expression was conducted through R package to identify key prognosis-associated genes in CC. To explore the relation between aberrant methylation and expression of genes, we extracted key genes with methylated expression from the downloaded data on TCGA CESC methylation. Then we evaluated the association between the methylated expression and the gene expression.

### Gene set enrichment analysis (GSEA)

According to the hub gene expression level, the samples were then separated into two different groups. To explore the potential function of the DEGs, GSEA [16] (http://software.broadinstitute.org/gsea/index.jsp) was used to research a series of biological pathways that might be enriched in the gene rank derived from hub gene among the two groups. Annotated gene set of c2.cp.kegg.v6.0.symbols.gmt in Molecular Signatures Database (MSigDB, http://software.broadinstitute.org/gsea/msigdb/index.jsp) was selected as the reference. Additionally, we used “Clusterprofiler” package in R to handle the datasets, and the “Enrichplot” package to tease out the enriched pathways of the key genes. The adjusted-*P* < 0.05 was set as significant.

## Results

### Identification of DEGs in CC

**Fig. 2:**
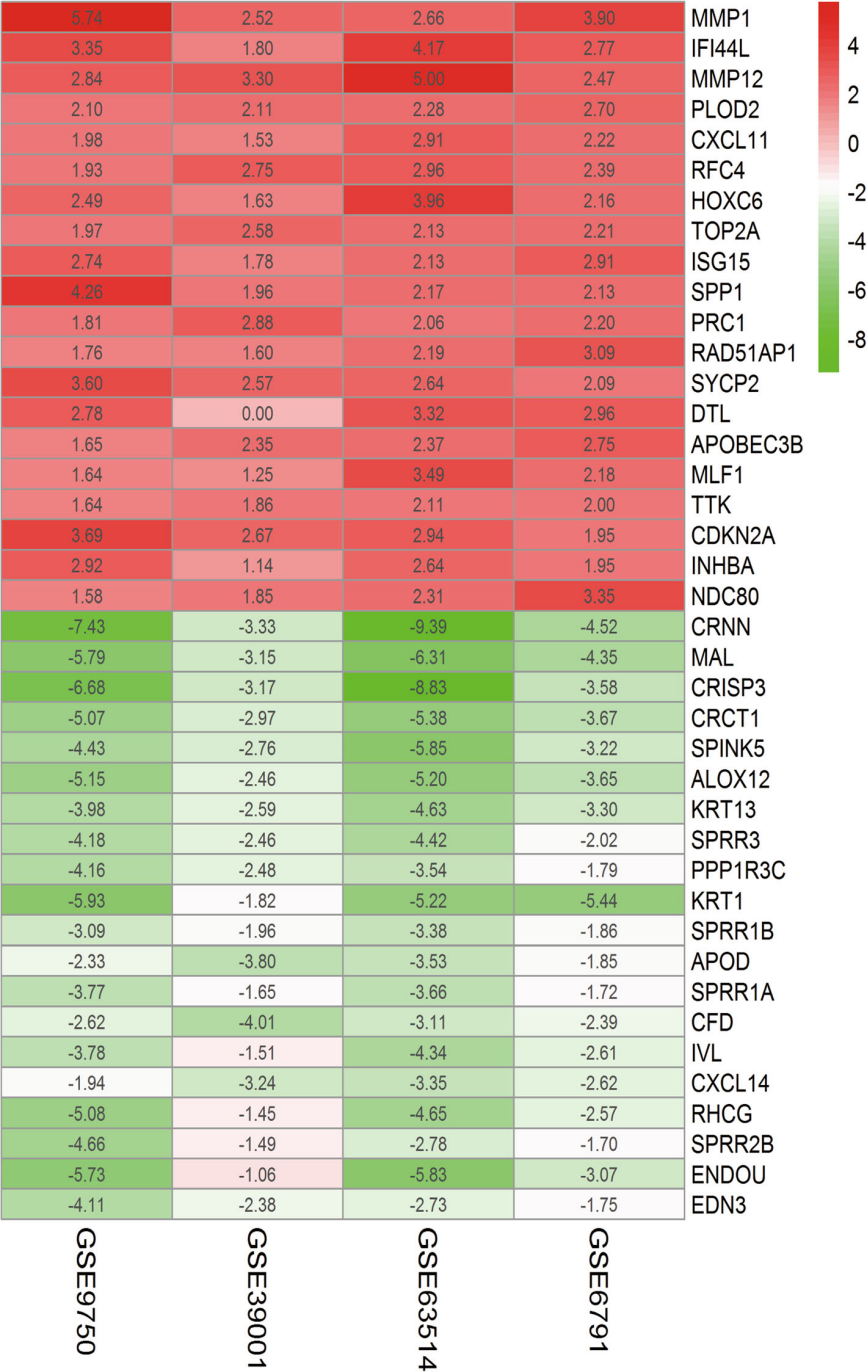
LogFC heatmap of the image data of each expression microarray. Notes: The abscissa is the geo ID, and the ordinate is the gene name. Red represents logFC>0, green represents logFC<0, and the values in the box represent the logFC values

The CC expression microarray datasets (GSE6791, GSE9750, GSE39001 and GSE63514) were firstly standardized (Figure S1). With limma package, 256 DEGs were filtered from GSE6791 (60 downregulated and 196 upregulated), 236 DEGs from GSE9750 (136 downregulated and 100 upregulated), 98 DEGs from GSE39001 (38 upregulated and 60 downregulated), 489 DEGs from GSE63514 (177 upregulated and 312 downregulated). DEGs from the 4 microarray datasets were exhibited in volcano maps (Figure S2A-D) and heatmaps (Figure S3A-D). We analyzed the four microarray datasets via the limma package and then with RRA method according to their log-folding variation values ((|log2fold change (FC)| > 1 and adj. *p* < 0.05). The RRA method was based on the theory that genes in each experiment were randomly ordered. For the genes ranking higher in the experiment, the possibility of differential expression is inversely proportional to the value of *P*. Through analytic hierarchy analysis, we sorted out 74 up-regulated and 73 down-regulated genes (Table 2). Finally, the R-heatmap software was performed to plot the top 40 up- and down-regulated genes (Fig. 2).

**Table 2:** Screening DEGs in cervical cancer by integrated microarray

DEGs	Gene name
Upregulated	MMP1 IFI44L MMP12 PLOD2 CXCL11 RFC4 HOXC6 TOP2A ISG15 SPP1 PRC1 RAD51AP1 SYCP2 DTL APOBEC3B MLF1 TTK CDKN2A INHBA NDC80 EZH2 CXCL8 KIF23 CTHRC1 MCM2 KIF20A KIF4A CDK1 MICB CENPE LAMP3 IFI44 CXCL13 CDC25B TOPBP1 CDC7 LMNB1 RRM2 CDC6 HLTF SYNGR3 NCAPG RYR1 ENO2 SMC4 NEK2 CXCL1 MCM3 C1QB SNX10 PPAP2C KIF11 MCM5 AIM2 AURKA MAD2L1 PBK CENPF KIF15 KNTC1 NTS FBXO5 STIL SPAG5 TRIP13 EPCAM MELK MMP3 KIF14 GZMB CDC20 CEP55 BUB1B NEFH
Downregulated	CRNN MAL CRISP3 CRCT1 SPINK5 ALOX12 KRT13 SPRR3 PPP1R3C KRT1 SPRR1B APOD SPRR1A CFD IVL CXCL14 RHCG SPRR2B ENDOU EDN3 CRYAB TMPRSS11D CLIC3 HPGD UPK1A TST KLK11 BBOX1 EMP1 CLCA4 KLK12 SCNN1B NSG1 SLURP1 SOSTDC1 IL1R2 KRT4 KLF4 DSG1 PPL DEFB1 SULT2B1 GPX3 TGM3 ALOX12B ECM1 NDN ISL1 CRABP2 FCGBP PTGDS TMPRSS11B CCND1 FOSB GYS2 TGFBR3 LDOC1 S100A7 KRT2 FGFBP1 PRSS3 ID4 ADRB2 VAT1 SLIT2 CLDN8 KLK10 PTK6 SPINK2 AR PDGFD AKR1B10 EREG

### Functional analysis of DEGs

**Fig. 3:**
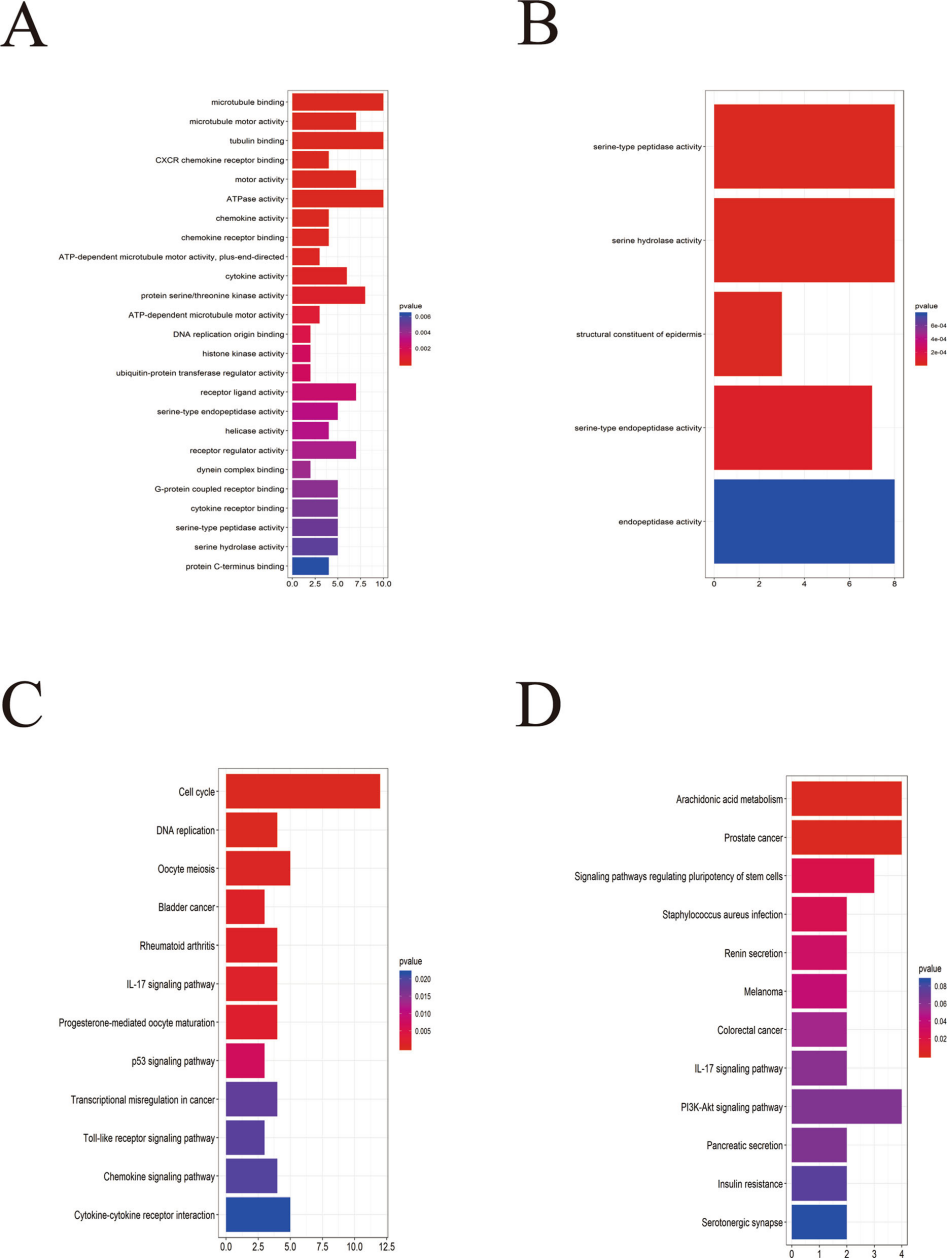
GO and KEGG analysis of the DEGs. **a** GO analysis of upregulated genes associated with cervical cancer. **b** GO analysis of downregulated genes associated with cervical cancer. **c** KEGG analysis of upregulated genes associated with cervical cancer. **d** KEGG analysis of downregulated genes associated with cervical cancer

The biological annotations of DEGs in CC were obtained with an online analysis tool named DAVID, which had GO analysis of up- and down-regulated genes (*P*<0.05). The GO analysis of DEGs covered three aspects: biological processes, molecular function and cellular composition (Figure S4A). The upregulated genes were significantly enriched in microtubule binding, tubulin binding and ATPase activity **(**Fig. 3a), and the down-regulated genes in serine-type peptidase activity, serine-type endopeptidase and serine hydrolase activity (Fig. 3b). These results indicated that most DEGs were prominently enriched in structural molecule activity, midbody, kinesin complex and microtubule motor activity. (Figure S4 B-C and Figure S5A). Clusterprofile was performed to analyze the DEGs. The result showed that the upregulated genes were mostly enriched in DNA replication, Oocyte meiosis and Cell cycle. (Fig. 3c), and the down-regulated genes in Arachidonic acid metabolism, prostate cancer and signaling pathways regulating pluripotency of stem cells (Fig. 3d). The pathway-gene network (Figure S5B) suggested that the cell cycle was the most important term in the biological processes of CC.

### PPI network construction and modules selection

**Fig. 4:**
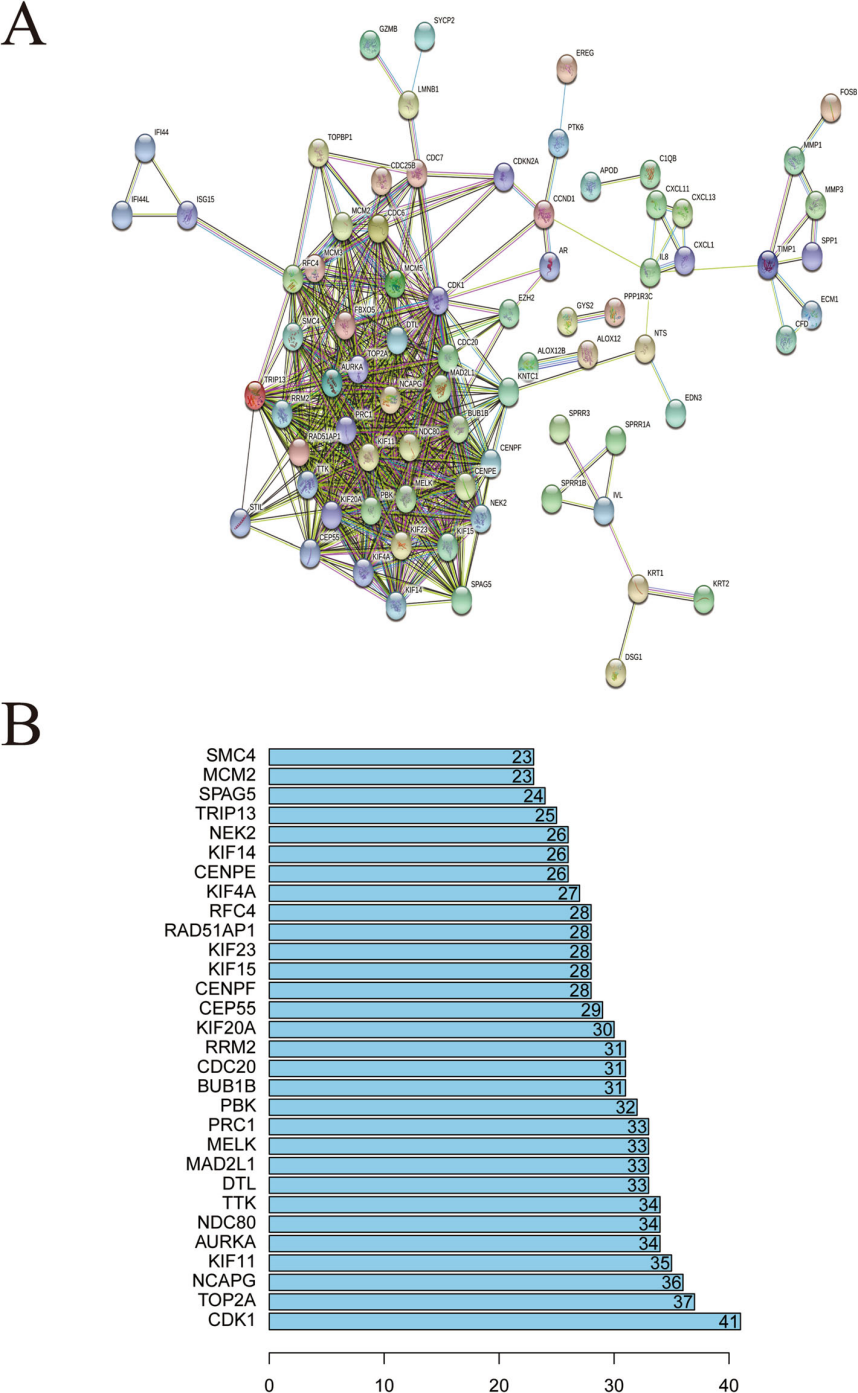
PPI network analysis. **a** Using the STRING online database, a total of 147 DEGs were filtered into the DEGs PPI network. **b** Degree of the top 30 nodes in the PPI network. All these nodes are upregulated genes

The PPI network of DEGs was constructed, including 147 nodes (74 up-regulated and 73 down-regulated genes) and 562 edges (Fig. 4a). Degrees ≥30 was set as the cutoff. A total of 16 genes, such as CDK1, TOP2A, NCAPG, and KIF11, were showed the most significant difference in expression (Fig. 4b). A significant module was selected with plug-in MCODE, including 27 nodes and 343 edges (Figure S6A). GO and KEGG analysis indicated that the genes in the module were related to microtubule binding, tubulin binding, cell cycle and oocyte mitosis (Figure S6B and C).

### Small molecule drugs screening

**Fig. 5:**
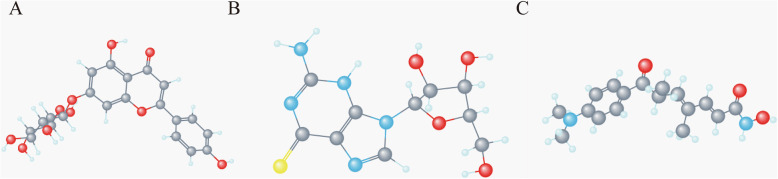
Three-dimensional diagram of the three most significant drugs. **a** Apigenin **b** Thioguanosine **c** Trichostatin A

CMap network was used to analyze 147 DEGs into two groups (74 in up-regulated group and 73 in down-regulated group). After the signature query, the three compounds with the highest negative enrichment score (apigenin, thioguanine, and trichostatin A) were identified as potential therapeutic agents for CC (Table 3). The three-dimensional chemical structure of these three compounds is shown in Fig. 5.

**Table 3:** Results of CMap analysis

Rank	CMap name	Mean	N	Enrichment	***P***-value
1	apigenin	−0.848	4	−0.973	0
2	thioguanosine	−0.799	4	−0.96	0
3	trichostatin A	−0.386	182	−0.261	0
4	viomycin	0.751	4	0.924	0.00004
5	adiphenine	0.779	5	0.907	0.00004
6	atractyloside	0.651	5	0.839	0.00024
7	chrysin	−0.745	3	−0.939	0.00032
8	isoflupredone	0.768	3	0.937	0.00044
9	nadolol	0.649	4	0.866	0.00044

### Validation of hub genes

**Fig. 6:**
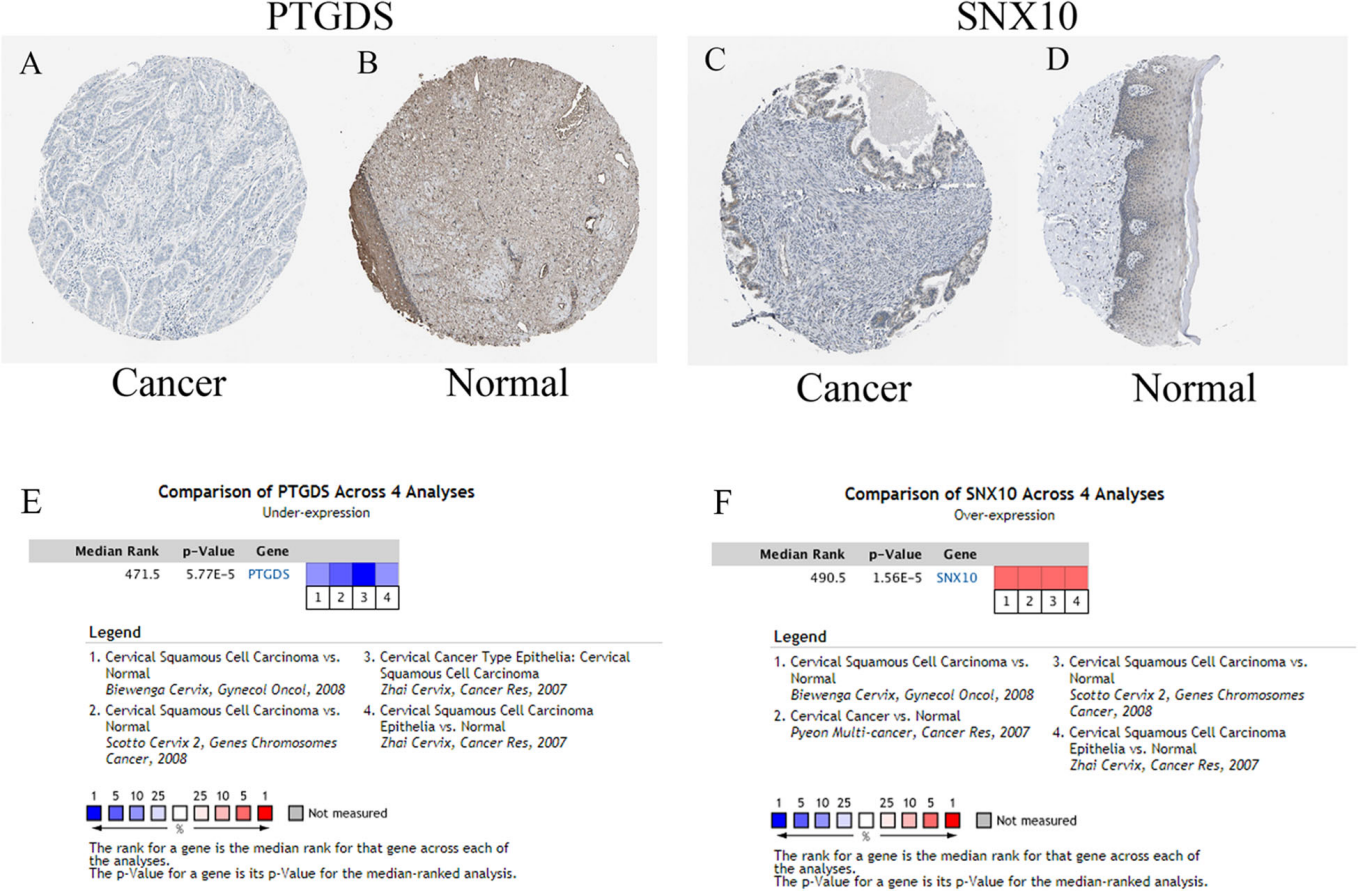
Validation of PTGDS and SNX10. **a** Immunohistochemistry of PTGDS. **b** Immunohistochemistry of SNX10. **c** Expression of PTGDS in ONCOMINE. **d** Expression of SNX10 in ONCOMINE

**Fig. 7:**
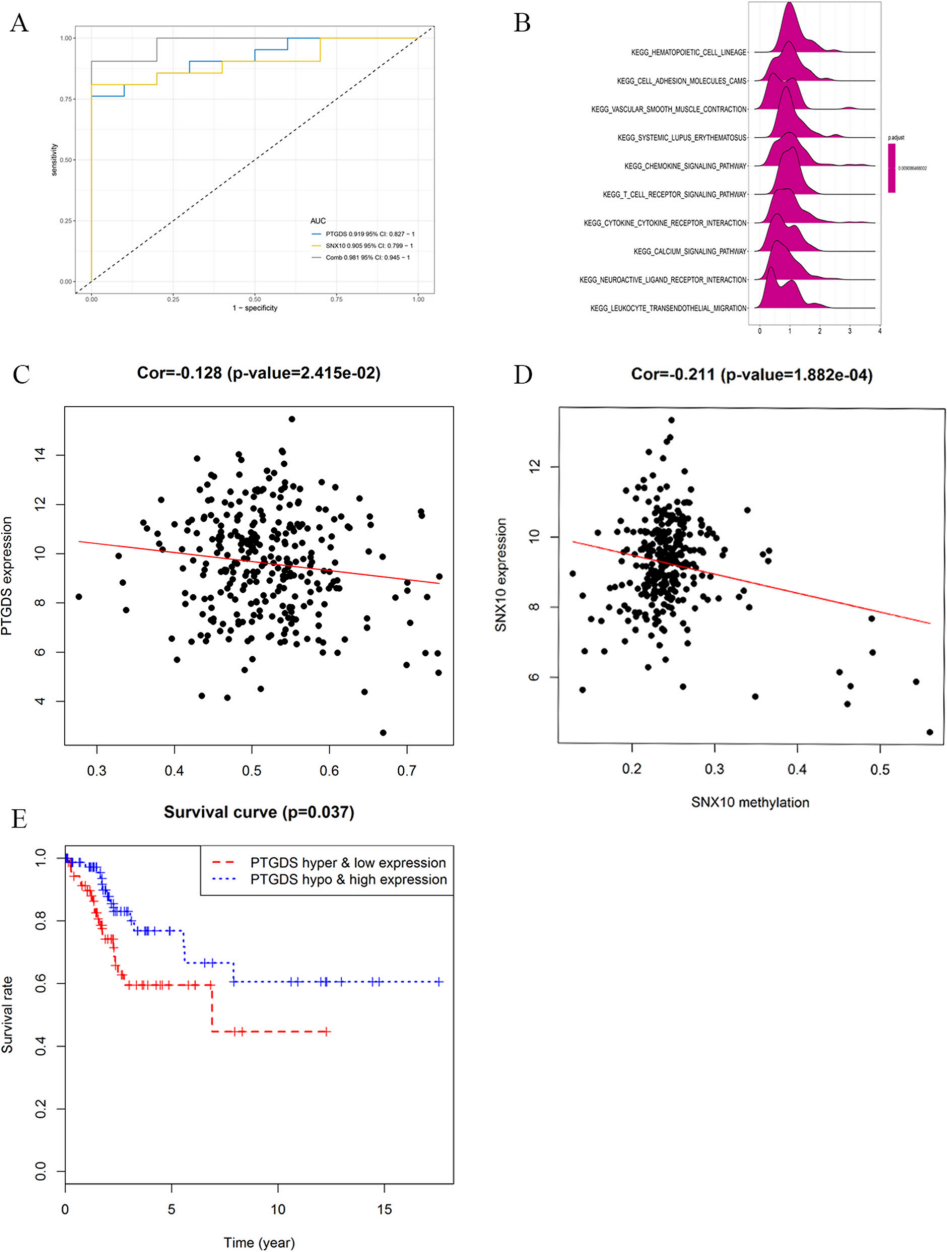
Validation of PTGDS and SNX10. **a** ROC curve analysis of the two genes. **b** GSEA of PTGDS and SNX10. **c** The methylated expression and gene expression of PTGDS. **d** The methylated expression and gene expression of SNX10. **e** Survival analysis of patients with methylated PTGDS expression

We validated DEGs at GEPIA website, including survival analysis and tissue sample expression analysis (Figure S7 and Figure S8). Eight genes (APOD, CXCL8, MMP1, MMP3, PLOD2, PTGDS, SNX10 and SPP1) had the same trend in both the above analysis. We literature-reviewed these eight genes, finding that only PTGDS and SNX10 had not been reported to be associated with CC. Therefore, we used GSE7803 and GSE29576 to validate PTGDS and SNX10 (Figure S9). The results showed that PTGDS had high expression levels in normal tissues and low expression levels in tumor tissues, while SNX10 showed an opposite profile. We further validated the two genes using immunohistochemistry (Fig. 6a-b) and ONCOMINE, obtaining the results consistent with those from the GEO database (Fig. 6c-d). The area under the curve of PTGDS was 0.919 and that of PTGDS was 0.905, suggesting that both can distinguish CC and normal tissue and have a good diagnostic efficiency (Fig. 7a). GSEA was performed to search KEGG pathways enriched in the TCGA samples. The top ten most enriched pathways included “hematopoietic cell lineage”, “adhesion molecules cams”, “vascular smooth muscle contraction”, “systemic lupus erythematosus”, “chemokine signaling pathway”, “t cell receptor signaling pathway”, “cytokine cytokine receptor interaction”, “calcium signaling pathway”, “neuroactive ligand receptor interaction” and “leukocyte transendothelial migration” (Fig. 7b) (adj.*p* < 0.05). In addition, the univariate and multivariate Cox regression analyses showed that SNX10 and PTGDS were independent prognostic indicators for OS among CESC patients (*P* = 0.007 and 0.003) (Table 4).

**Table 4:** Univariate analysis and multivariate analysis of SNX10 and PTGDS expression among cervical cancer patients

Variables	Univariate analysis			Multivariate analysis		
	HR	95%CI	*p*	HR	95%CI	*p*
Stage (I & II) vs Stage (III & IV)	2.338	1.364–4.004	**0.002**	3.078	1.329–7.129	**0.009**
Grade(G1 & G2) vs Grade(G3)	1.221	0.947–1.574	0.124	0.837	0.565–1.240	0.375
Age(≤50) vs Age(> 50)	1.263	0.755–2.112	0.373	1.197	0.711–2.011	0.498
SNX10 (high expression) vs SNX10 (low expression)	1.202	0.957–1.509	0.113	1.424	1.103–1.838	**0.007**
PTGDS (high expression) vs PTGDS (low expression)	0.838	0.729–0.962	**0.012**	0.802	0.693–0.928	**0.003**

To find out the mechanism of abnormal gene expression, we analyzed the gene expression level and methylation level from the Illumina Human Methylation 450 platform based on TCGA data. The association between the methylated expression and the gene expression of the two key driving genes were shown in Fig. 7c-d. The survival analysis showed that the patients with low-expressed and hyper-methylated PTGDS had a worse prognosis than those with high-expressed and hypo-methylated PTGDS (*P* = 0.037) (Fig. 7e). However, SNX10 methylation has no statistical significance in survival analysis.

### Establishment of cox regression model

**Fig. 8:**
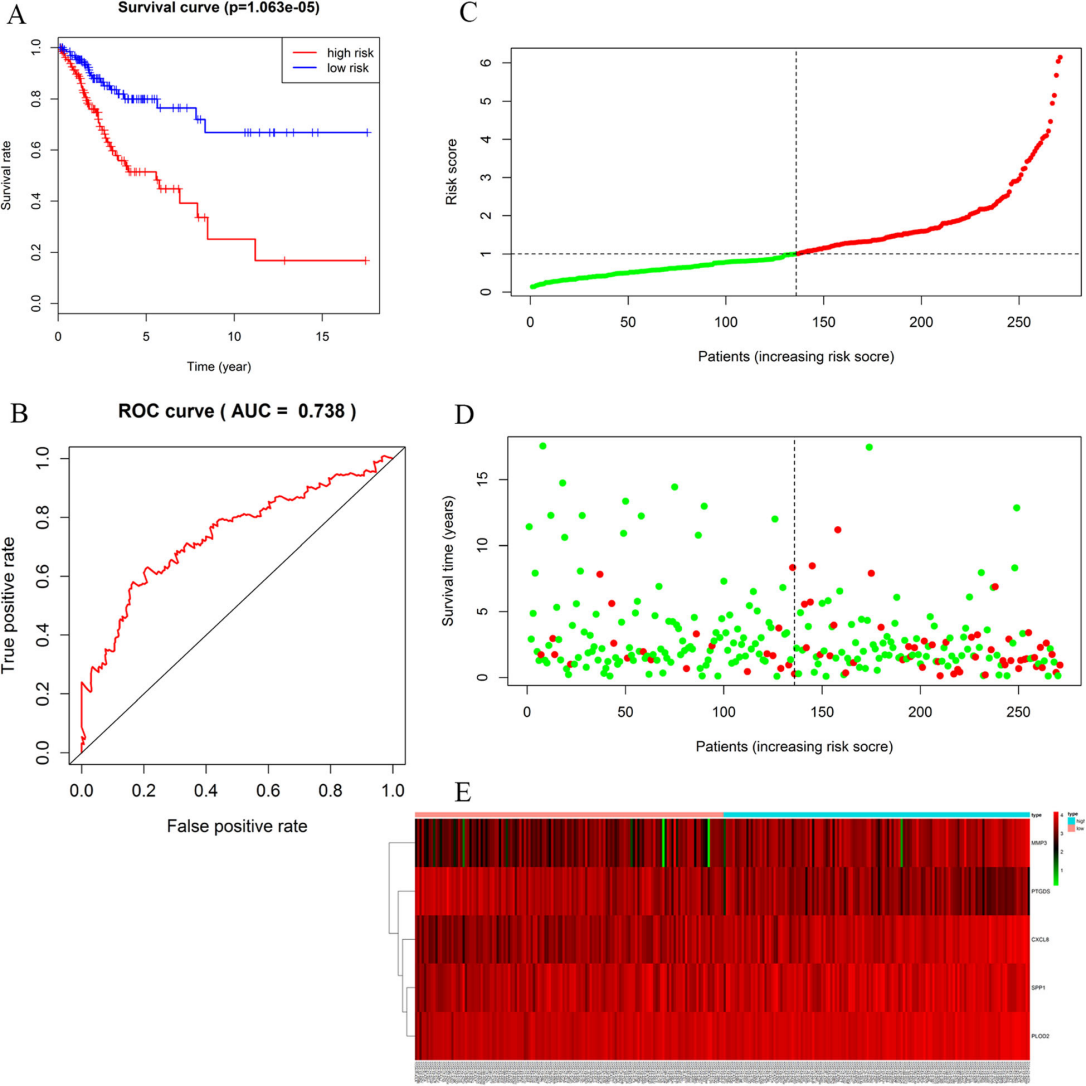
Survival prognosis model of the 5 hub genes. **a** Survival analysis showed that the patients in the high risk group had statistically significantly worse overall survival than those in low risk group in TCGA cohort. **b** ROC analysis was performed to find out the most optimal cutoff value to divide the CC patients into high risk and low risk group. **c-d** The risk scores for all patients in TCGA cohort are plotted in ascending order and marked as low risk (blue) or high risk (red), as divided by the threshold (vertical black line). **e** Eight expression and risk score distribution in TCGA cohort by z-score, with red indicating higher expression and light blue indicating lower expression

Univariate cox regression analysis screened out seven genes, including APOD, CXCL8, MMP1, MMP3, PLOD2, PTGDS and SPP1 (Figure S10). Multivariate Cox regression analysis screened five genes, including SPP1, CXCL8, PTGDS, PLOD2 and MMP3 (Figure S11). The score for overall survival risk prediction was calculated as followed: Risk score = 0.143* SPP1 + 0.136* CXCL8–0.093* PTGDS+ 0.206* PLOD2 + 0.067* MMP3. Based on the risk score, CC patients were divided into low- and high-risk groups. Kaplan-Meier analysis suggested that low-risk patients had better outcomes than high-risk patients in the TCGA cohort (Fig. 8a). ROC curve analysis was also completed according to the 5-year survival of the area under the receiver operating characteristic curve (AUC) value. The specificity and sensitivity were both highest when the risk score was 0.738 (Fig. 8b). The distribution of risk score, survival status, and the expression levels of five genes was also presented (Fig. 8c-f). The expression levels of five genes in low- and high-risk groups were shown in Figure S12A. The univariate and multivariate Cox regression analyses showed that only the risk score based on five genes was independent prognostic indictor of CC (Figure S12B-C).

The Heatmap showed the expression levels of the five genes in two subgroup patients in the TCGA dataset. We observed significant difference in survival state (*P* < 0.001) and stage (*P* < 0.05) (Figure S12D).

## Discussion

CC brings on more than 265,700 deaths per year, making it the second deadlist malignancy in women. Nowadays, microarray and high-throughput sequencing technology are used to identify the potential targets in CC treatment. Previous studies often establish a single group or have a small-size, which restricts their reliability.

This study analyzed the expression profiles of four genes using R software and bioinformatics tools. A total of 147 differential genes were identified using RRA analysis, including 73 downregulated and 74 upregulated. GO analysis indicated that upregulated DEGs were associated with microtubule binding and downregulated DEGs with serine-type peptidase activity. KEGG analysis showed that these DEGs were primarily enriched in the cell cycle pathway.

Our findings echo with the previous studies. It has been reported that microtubule binding and cell cycle have an effect on CC [17]. Other studies showed that microtubule binding played a role in the biology of acute myeloid leukemia cells and colorectal cancer cells [18, 19]. Cell cycle also decides the abnormal proliferation of many tumor cells [20, 21].

PPI network displayed 30 hub genes associated with CC associated proteins. Next, we found CDK1 was in the center of PPI network of CESC. CDK1 was harbored by top module 1, suggesting that the top module 1 plays a crucial role in CC pathogenesis. Functional analysis indicated that the genes in this module were mainly enriched in microtubule binding, tubulin binding and cell cycle. Cyclin-dependent kinase 1 (CDK1) is a serine/threonine kinases that interacted with specific cyclins to regulate the cell cycle [22]. It has been reported that CDK1 regulated the development of CC and many other tumors [23]. Y. Zeng et al. found that the mitotic phosphorylation level of the transcriptional co-repressor Vgll4 was mediated by CDK1 to its tumor-suppressing activity. K. Bednarek et al. found that CDK1 was involved in the processes of laryngeal squamous cell carcinoma [24, 25].

Cmap showed that apigenin, thioguanine and trichostatin A could be used to treat CC. Apigenin and trichostatin A can inhibit breast cancer growth [26, 27]. 6-thioguanine has also potential therapeutic effects on tumors [28]. Our findings may help create the appropriate drugs for CC treatment.

Eight genes (APOD, CXCL8, MMP1, MMP3, PLOD2, PTGDS, SNX10 and SPP1) were screened out of DEGs through GEPIA. Of them, only PTGDS and SNX10 had not been reported in CC research. According to GEO validation results, PTGDS was lowly expressed and SNX10 highly expressed in tumor tissues, which was consistent with the results from immunohistochemistry. TCGA methylation analysis showed that the patients with lowly-expressed and highly-methylated PTGDS had a worse prognosis than those with highly-expressed and lowly-methylated PTGDS. Cox regression analysis showed that SNX10 and PTGDS were independent prognostic indicators for OS among CC patients. GSEA showed that the two genes were associated with the chemokine signaling pathway. Zhong G et al. suggested that chemokine signaling is involved in the invasion and migration of lung cancer cells [29]. Chemokine signaling has also been reported to maneuver the progression of breast and hepatobiliary cancer [30, 31]. In addition, the prognostic signature was constructed based on the eight hub genes. Of them, five genes (SPP1, CXCL8, PTGDS, PLOD2, and MMP3) exhibited significant prognostic value. The Cox regression analysis showed that only the risk score of the five genes was an independent prognostic indicator of CC.

Interestingly, all of the above genes are associated with cervical cancer. Yan R et al. found that CXCL8 had prognostic value in cervical carcinoma patients [32]. Tian R et al. identified the function of MMP1 in cervical cancer [33]. Xie B et al. defined that genetic polymorphisms in MMP 3 connected with the clinical outcome of cervical cancer in a Chinese Han population [34]. Xu F et al. found that increased PLOD2 expression facilitated epithelial-to-mesenchymal transition (EMT) and focal adhesion formation, thus promoting the migration and invasion of cervical cancer cells [35]. Chen X et al. found SPP1 inhibition enhanced the chemosensitivity of cervical cancer cell lines to cisplatin [36]. Using microarray analysis, Song JY et al. found that APOD played in the invasion of cervical cancer [37]. These genes play different roles in other tumors. For example, Allina DO et al. demonstrated the diagnostic significance of APOD for prostatic neoplasms [38]. Shen T et al. held that CXCL8 induced EMT in colon cancer [39]. Wang Y et al. found that CXCL8 regulated the development of breast cancer [40]. Ha H et al. found that CXCL8 was also involved in inflammatory diseases in addition to tumors [41]. Liu M et al. argued that MMP1 promoted the growth and metastasis of esophageal squamous cell carcinoma [42]. MMP1 also participated in breast and ovarian cancer [43, 44]. Banik D et al. demonstrated that MMP3 regulated tumor progression [45]. Ji Y et al. demonstrated that C/EBPβ promoted tumor cell invasion and metastasis of colorectal cancer [46]. PLOD2 is implicated in cervical cancer [35] and renal cell carcinoma [47]. SPP1 linked with lung adenocarcinoma, gastric cancer and colorectal cancer [48–50].

Sorting nexin 10 (SNX10) can suppress the progression of colorectal cancer, liver cancer and stomach cancer [51, 52]. Cervantes-Anaya N et al. demonstrated SNX10/V-ATPase pathway regulated ciliogenesis in vitro and in vivo [52]. Prostaglandin D synthase (PTGDS) has also been intensely studied [53]. Omori K et al. demonstrated that PTGDS attenuated the malignance of tumor endothelial cells and regulated the processes in non-small cell lung cancer and gastric cancer [54, 55]. The present study is the first to report the expression and prognostic calue of these two genes in CC. Their methylation is associated with CC prognosis, a finding that has never been reported before.

This study has some limitations. First, the analysis is entirely based on open databases, so its results should be validated with functional experiments. Second, the five genetic profiles are based on a single cohort with relatively small sample size. Further studies should involve larger independent cohorts.

## Conclusion

Our study indicated that two novel genes PTGDS and SNX10 showed abnormal expression and methylation associated with CC development and explored their prognostic value. However, biofunctions of two genes remained to be unveiled with more in-depth research. The two genes might serve as potential prognostic biomarkers and therapeutic targets in the treatment of CC.

## Supplementary Information

**Additional file 1: Figure S1**. Standardization of gene expression. The blue bar represents the data before normalization, and the red bar represents the normalized data.(A) The standardization of GSE6791 data, (B) the standardization of GSE9750 data, (C) the standardization of GSE39001 data (D) the standardization of GSE63514 data.

**Additional file 2: Figure S2**. Volcanic maps of DEGs from all samples. The red dots represent upregulated genes screened according to |fold change| ≥ 2.0 and a corrected *P*-value ≤0.05. The green points represent downregulated genes and the cutoff value is the same as that of upregulated genes. The black points represent genes with no significant difference. (A)GSE6791 data, (B) GSE9750 data, (C) GSE39001 data and (D) GSE63514 data.

**Additional file 3: Figure S3**. Heatmap of DEGs from 4 datasets. Red cubes represent upregulated genes, green cubes represent downregulated genes, and black represent genes with no significance. Genes underexpressed are painted with gray. DEGs are screened by criterion:|fold change| ≥ 2.0 and a corrected *P*-value ≤0.05. (A) GSE6791 data, (B) GSE9750 data, (C) GSE39001 data and (D) GSE63514 data.

**Additional file 4: Figure S4**. GO enrichment analysis of DEGs in cervical cancer. (A) GO analysis divided DEGs into three functional groups: molecular function, biological processes, and cell composition. (B) The top 10 GO terms of DEGs in CC. The outer circle shows a scatter plot for each term of the logFC of the assigned genes. Red circles display upregulation and blue ones downregulation.(C) Distribution of DEGs in cervical cancer for different GO-enriched functions.

**Additional file 5: Figure S5.** (A) Collections between significant GO groups and DEGs. Blue rounds represent the GO groups, green rounds represent downregulated genes and red rounds represent upregulated genes. (B) Collections between significant KEGG groups and DEGs. Blue rounds represent signaling pathway, green rounds represent downregulated genes and red rounds represent upregulated genes.

**Additional file 6: Figure S6**. (A) A significant module selected from protein–protein interaction network. (B) GO analysis of these significant molecule.(C) KEGG analysis of these significant molecule.

**Additional file 7: Figure S7.** Validation of genes expression in GEPIA. (A) APOD, (B) CXCL8, (C) MMP1, (D) MMP3, (E) PLOD2, (F) PTGDS, (G) SNX10, (H) SPP1.

**Additional file 8: Figure S8.** Survival analysis of genes in GEPIA. (A) APOD, (B) CXCL8, (C) MMP1, (D) MMP3, (E) PLOD2, (F) PTGDS, (G) SNX10, (H) SPP1.

**Additional file 9: Figure S9.** Gene expression in GSE7803 and GSE29570. (A) PTGDS in GSE7803. (B) SNX10 in GSE7803. (C) PTGDS in GSE29570. (D) SNX10 in GSE29570.

**Additional file 10: Figure S10.** Seven genes were screened by Univariate cox regression analysis.

**Additional file 11: Figure S11.** Five genes were screened by Multivariate cox regression analysis.

**Additional file 12: Figure S12.** Regression analysis of the 5 genes identified. (A) The expression level of the 5 genes in low- and high-risk groups. (B) The univariate Cox proportional hazards regression. (C) The multivariate Cox proportional hazards regression. (D) The heatmap of the 5 genes in high- and low-risk patients in TCGA dataset.

## Data Availability

The datasets used and/or analysed during the current study are available from the corresponding author on reasonable request.

## Abbreviations

CC: Cervical cancer
DEGs: Differentially expressed gene
GEO: Gene Expression Omnibus
RRA: RobustRankAggreg
GO: Gene Ontology
KEGG: Kyoto Encyclopedia of Genes and Genomes
Cmap: Connectivity Map
PPI: Protein–protein interaction
STRING: Search Tool for the Retrieval of Interacting Genes Database
MCODE: Molecular Complex Detection
GEPIA: Gene Expression Profiling Interactive Analysis
ROC: Receiver operating characteristic
GSEA: Gene set enrichment analysis
