# Supplementary figures and images for: SNX10 and PTGDS are associated with the progression and prognosis of cervical squamous cell carcinoma
Source: BMC Cancer. 2021 Jun 11;21:694. doi: 10.1186/s12885-021-08212-w (PMC8196508; doi:10.1186/s12885-021-08212-w)

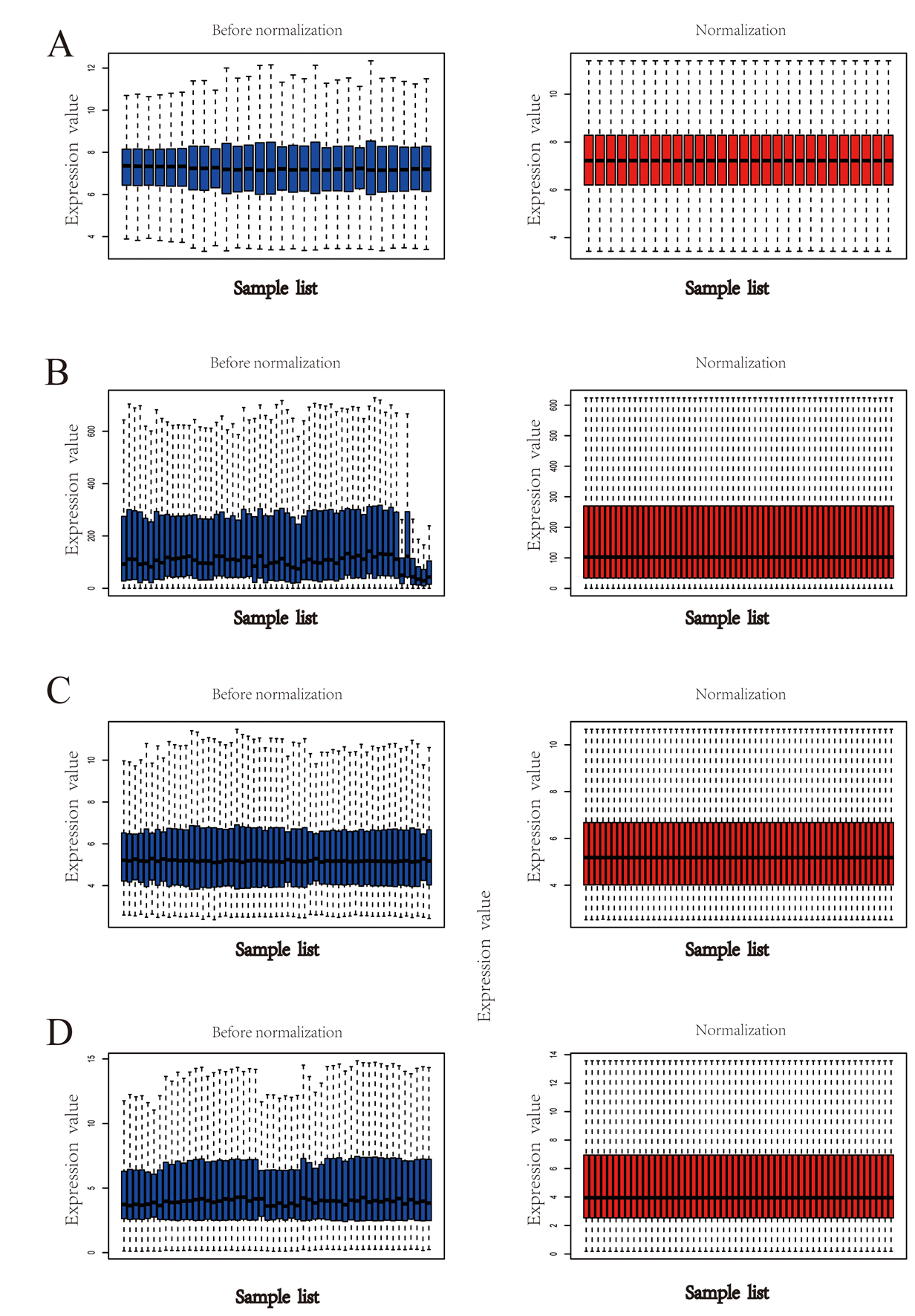

Supplement: Supplementary file 1 — Additional file 1: Figure S1. Standardization of gene expression. The blue bar represents the data before normalization, and the red bar represents the normalized data.(A) The standardization of GSE6791 data, (B) the standardization of GSE9750 data, (C) the standardization of GSE39001 data (D) the standardization of GSE63514 data. [file 12885_2021_8212_MOESM1_ESM.tif]

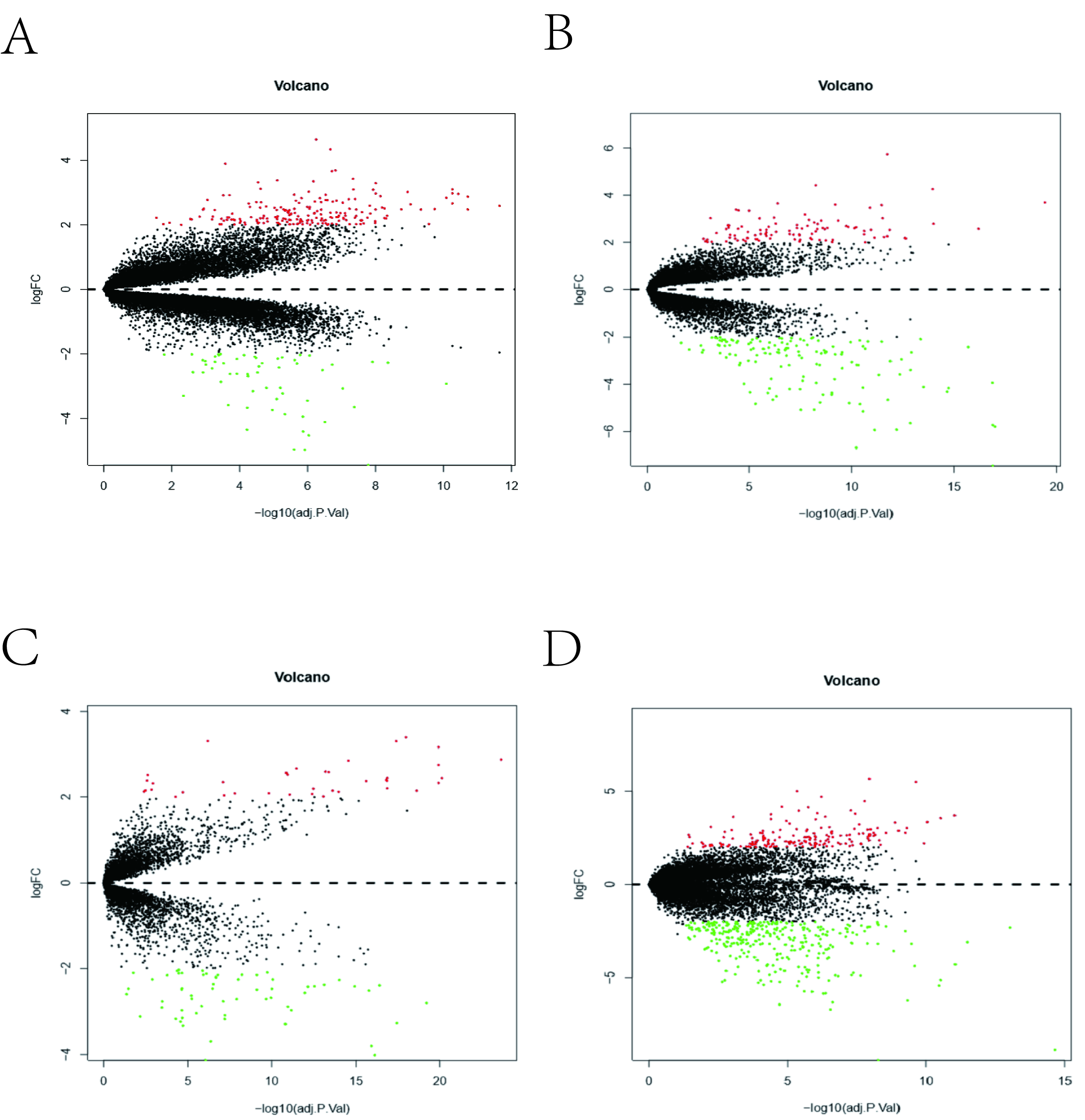

Supplement: Supplementary file 2 — Additional file 2: Figure S2. Volcanic maps of DEGs from all samples. The red dots represent upregulated genes screened according to |fold change| ≥ 2.0 and a corrected P-value ≤0.05. The green points represent downregulated genes and the cutoff value is the same as that of upregulated genes. The black points represent genes with no significant difference. (A)GSE6791 data, (B) GSE9750 data, (C) GSE39001 data and (D) GSE63514 data. [file 12885_2021_8212_MOESM2_ESM.tif]

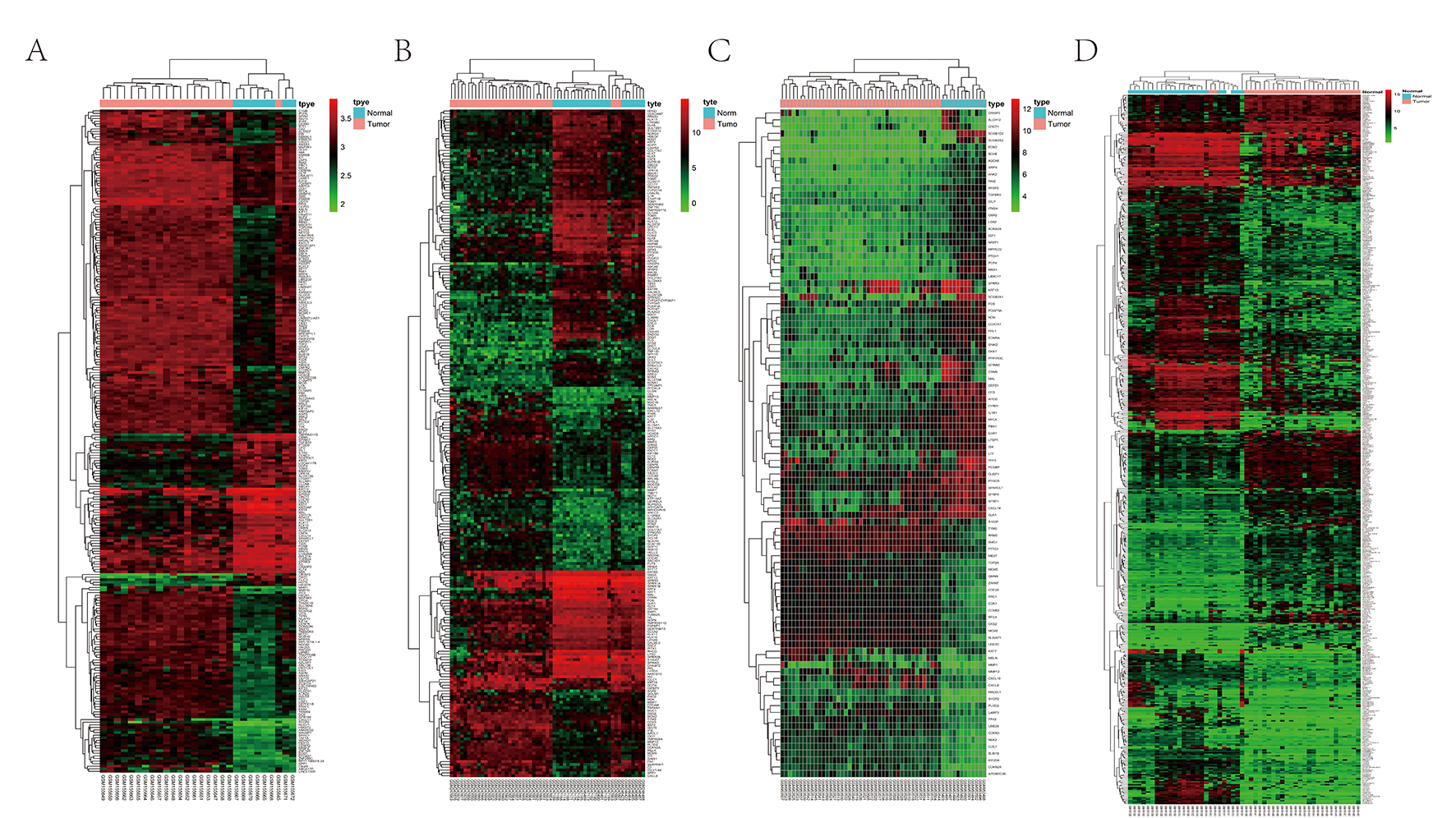

Supplement: Supplementary file 3 — Additional file 3: Figure S3. Heatmap of DEGs from 4 datasets. Red cubes represent upregulated genes, green cubes represent downregulated genes, and black represent genes with no significance. Genes underexpressed are painted with gray. DEGs are screened by criterion:|fold change| ≥ 2.0 and a corrected P-value ≤0.05. (A) GSE6791 data, (B) GSE9750 data, (C) GSE39001 data and (D) GSE63514 data. [file 12885_2021_8212_MOESM3_ESM.tif]

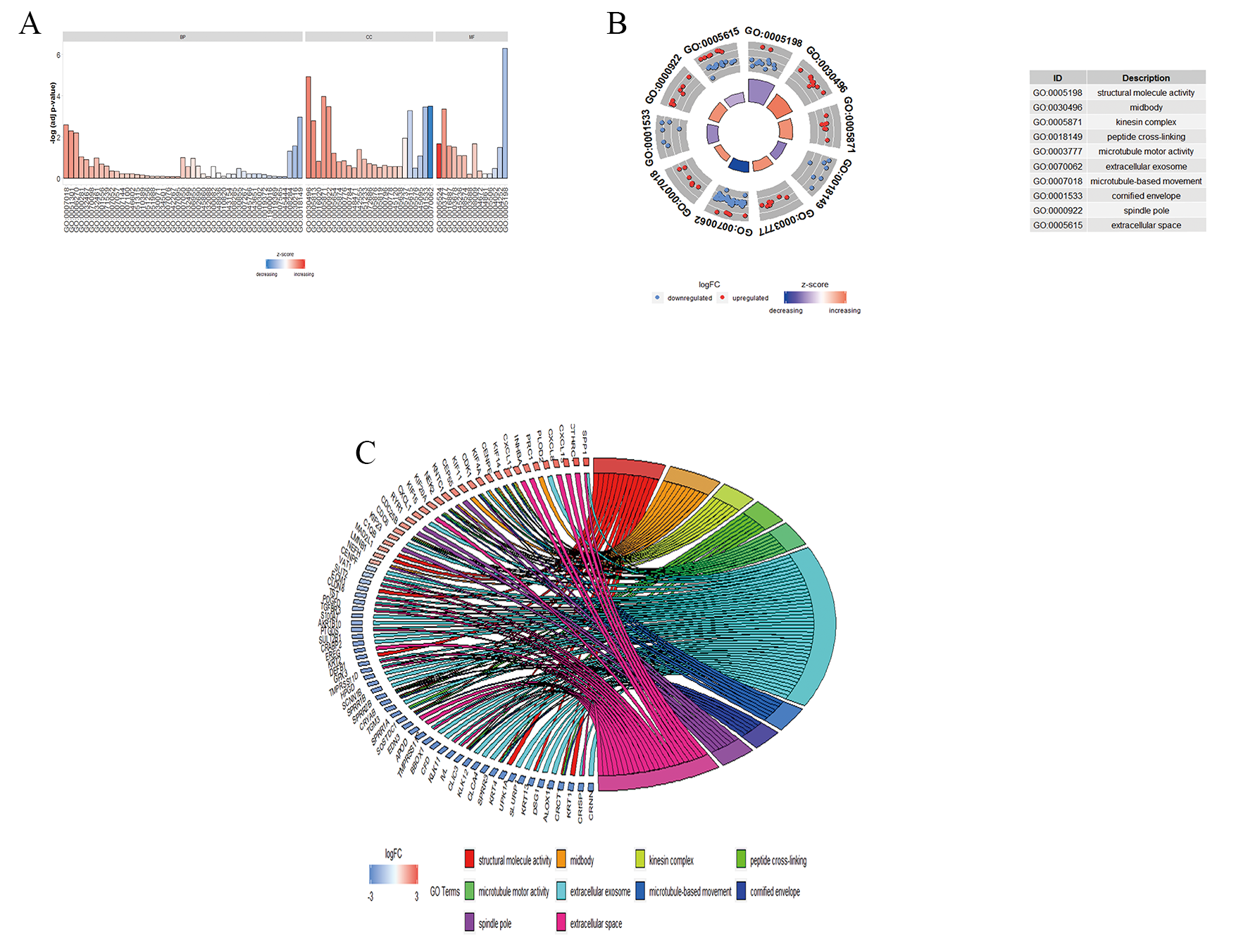

Supplement: Supplementary file 4 — Additional file 4: Figure S4. GO enrichment analysis of DEGs in cervical cancer. (A) GO analysis divided DEGs into three functional groups: molecular function, biological processes, and cell composition. (B) The top 10 GO terms of DEGs in CC. The outer circle shows a scatter plot for each term of the logFC of the assigned genes. Red circles display upregulation and blue ones downregulation.(C) Distribution of DEGs in cervical cancer for different GO-enriched functions. [file 12885_2021_8212_MOESM4_ESM.tif]

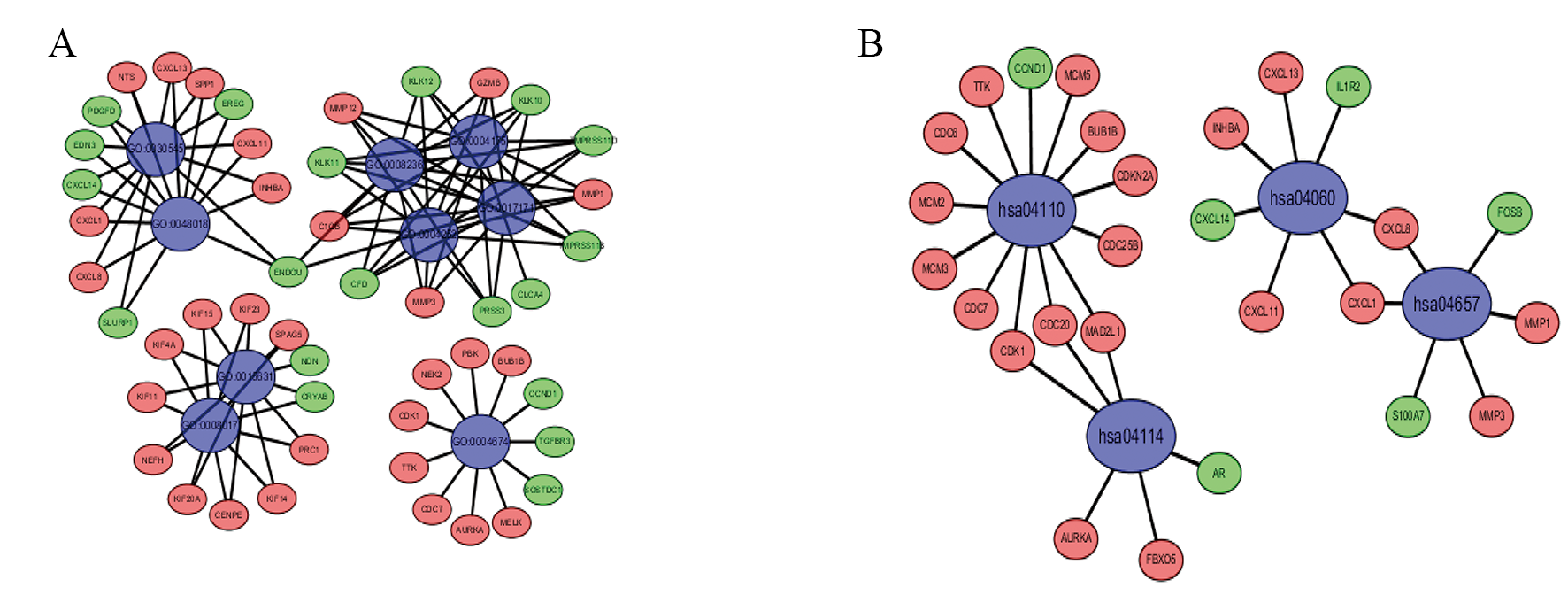

Supplement: Supplementary file 5 — Additional file 5: Figure S5. (A) Collections between significant GO groups and DEGs. Blue rounds represent the GO groups, green rounds represent downregulated genes and red rounds represent upregulated genes. (B) Collections between significant KEGG groups and DEGs. Blue rounds represent signaling pathway, green rounds represent downregulated genes and red rounds represent upregulated genes. [file 12885_2021_8212_MOESM5_ESM.tif]

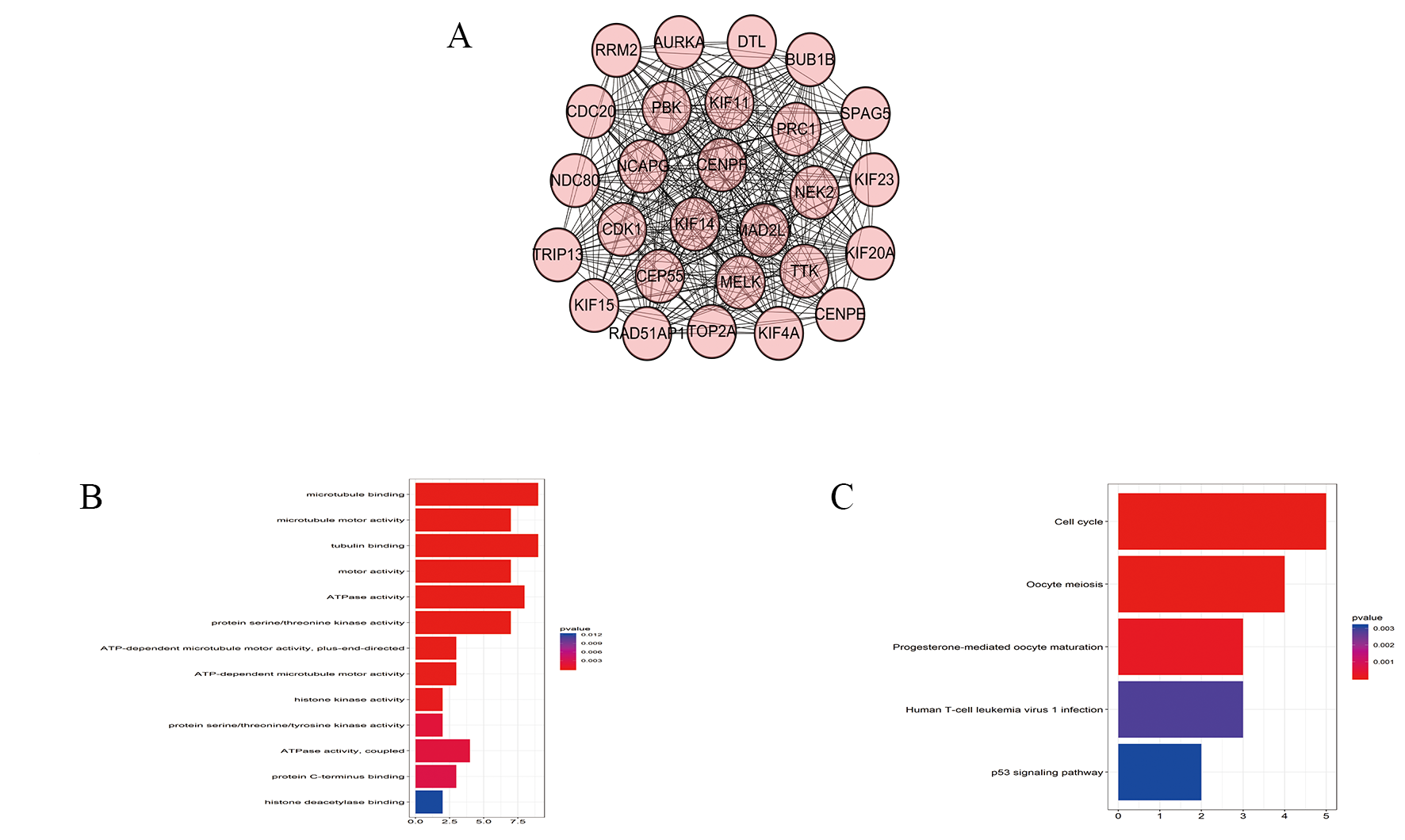

Supplement: Supplementary file 6 — Additional file 6: Figure S6. (A) A significant module selected from protein–protein interaction network. (B) GO analysis of these significant molecule.(C) KEGG analysis of these significant molecule. [file 12885_2021_8212_MOESM6_ESM.tif]

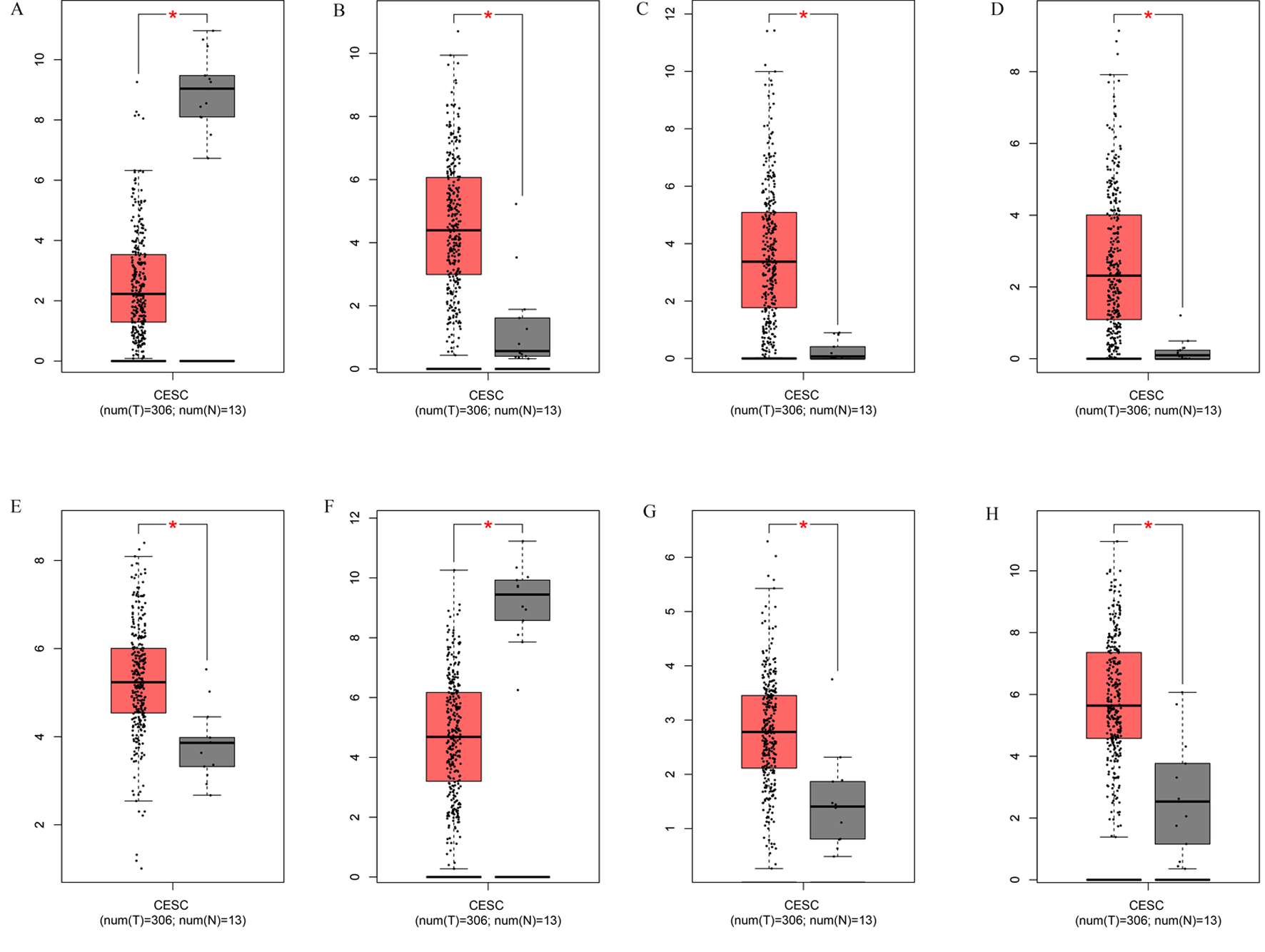

Supplement: Supplementary file 7 — Additional file 7: Figure S7. Validation of genes expression in GEPIA. (A) APOD, (B) CXCL8, (C) MMP1, (D) MMP3, (E) PLOD2, (F) PTGDS, (G) SNX10, (H) SPP1. [file 12885_2021_8212_MOESM7_ESM.tif]

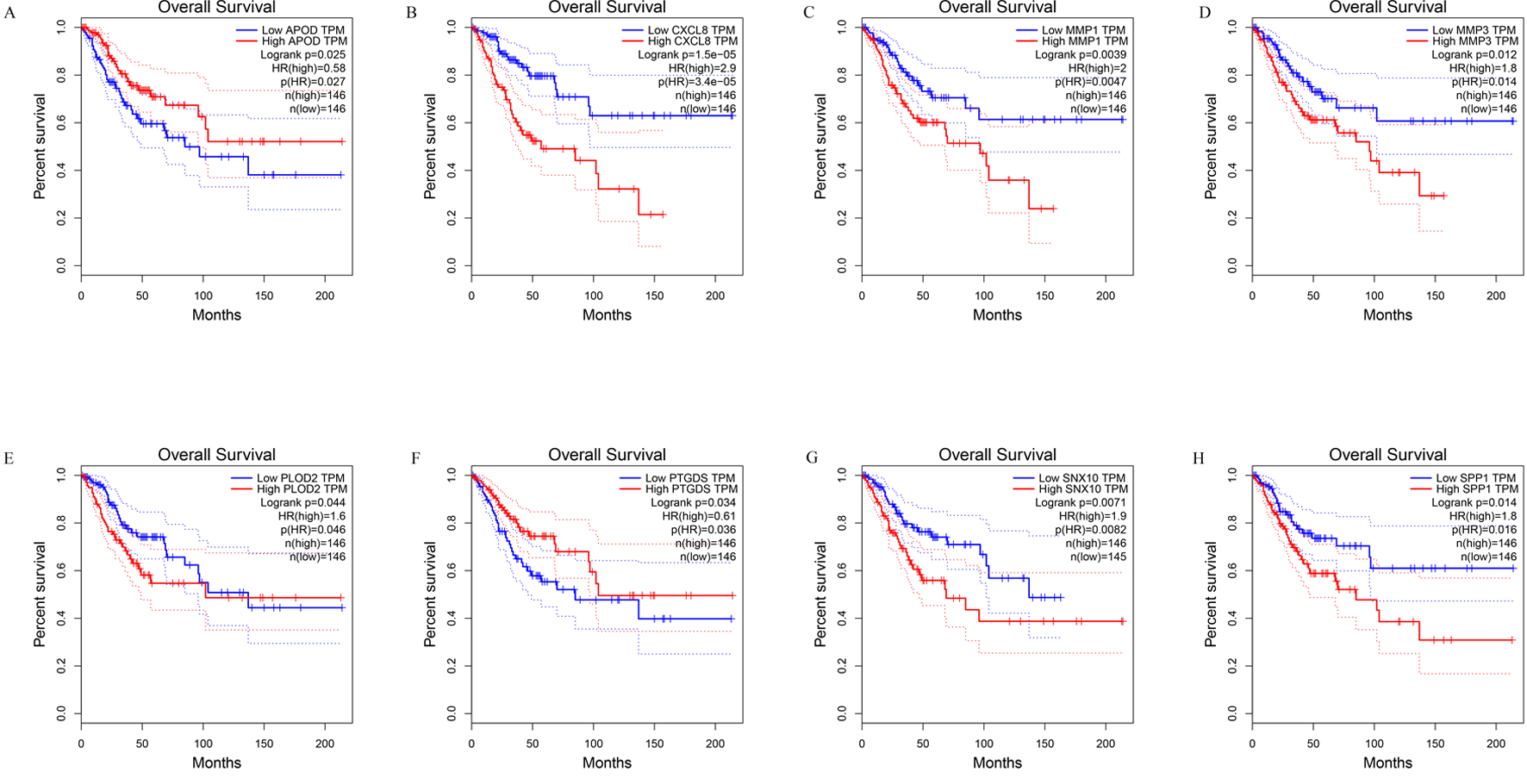

Supplement: Supplementary file 8 — Additional file 8: Figure S8. Survival analysis of genes in GEPIA. (A) APOD, (B) CXCL8, (C) MMP1, (D) MMP3, (E) PLOD2, (F) PTGDS, (G) SNX10, (H) SPP1. [file 12885_2021_8212_MOESM8_ESM.tif]

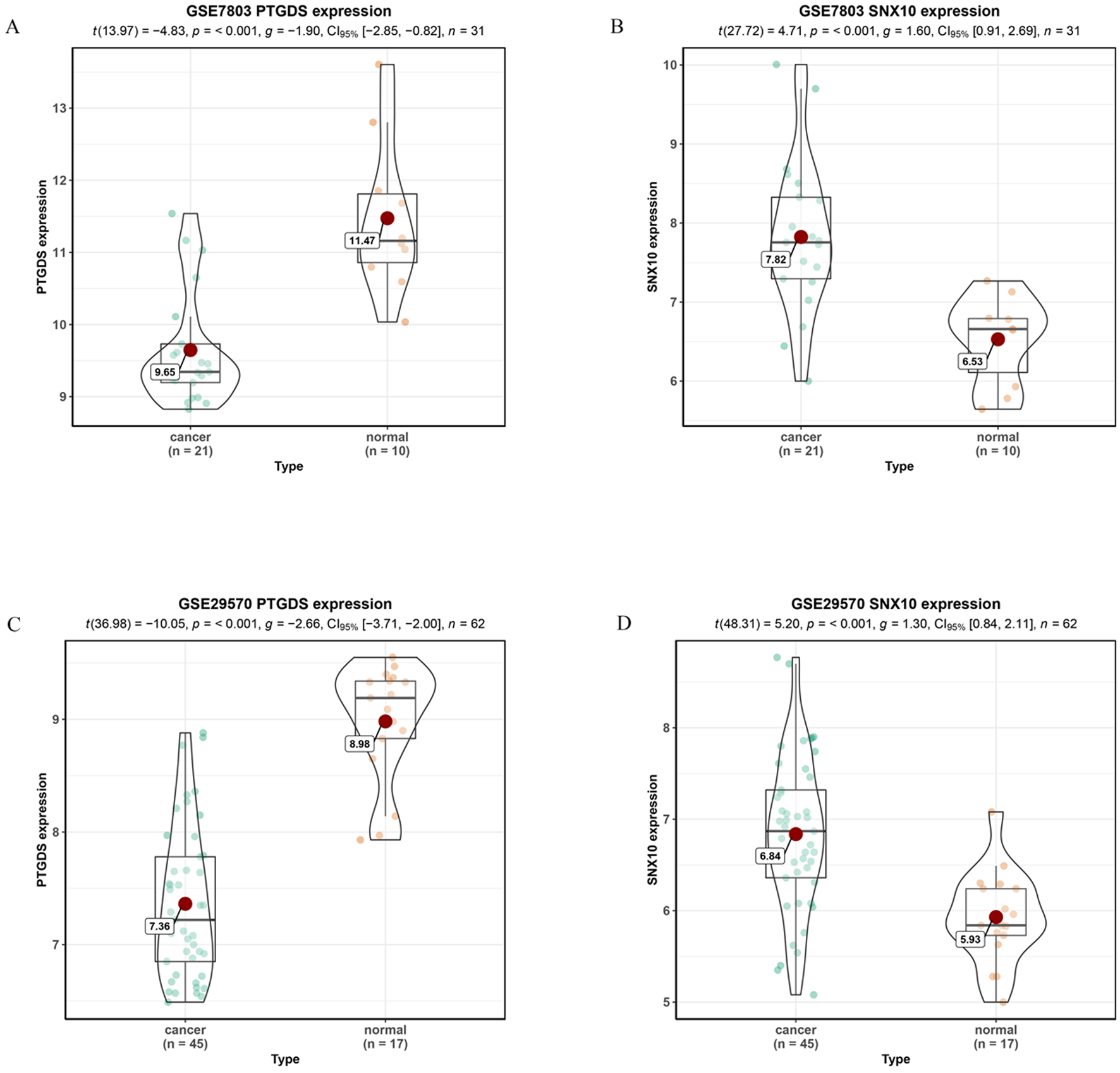

Supplement: Supplementary file 9 — Additional file 9: Figure S9. Gene expression in GSE7803 and GSE29570. (A) PTGDS in GSE7803. (B) SNX10 in GSE7803. (C) PTGDS in GSE29570. (D) SNX10 in GSE29570. [file 12885_2021_8212_MOESM9_ESM.tif]

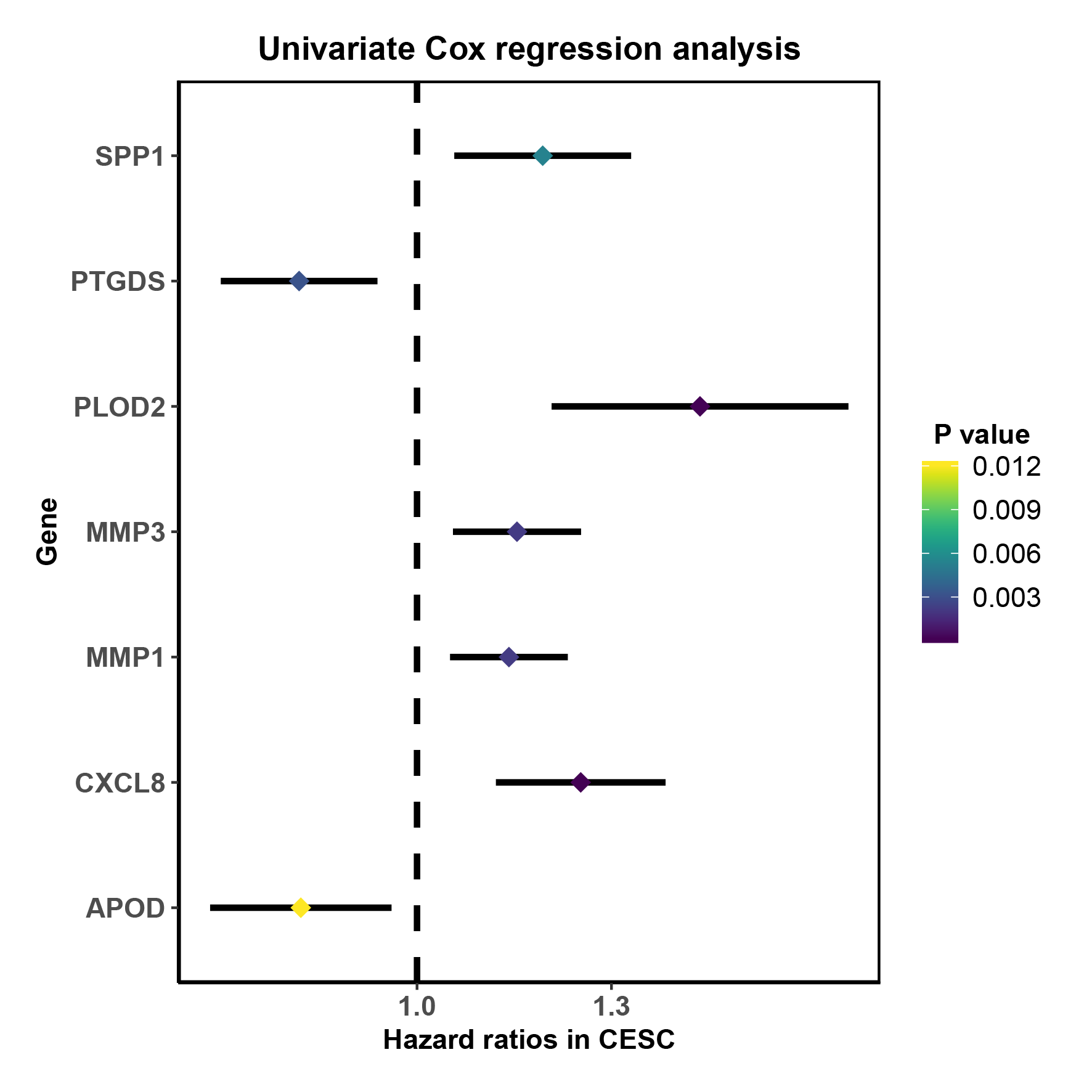

Supplement: Supplementary file 10 — Additional file 10: Figure S10. Seven genes were screened by Univariate cox regression analysis. [file 12885_2021_8212_MOESM10_ESM.tif]

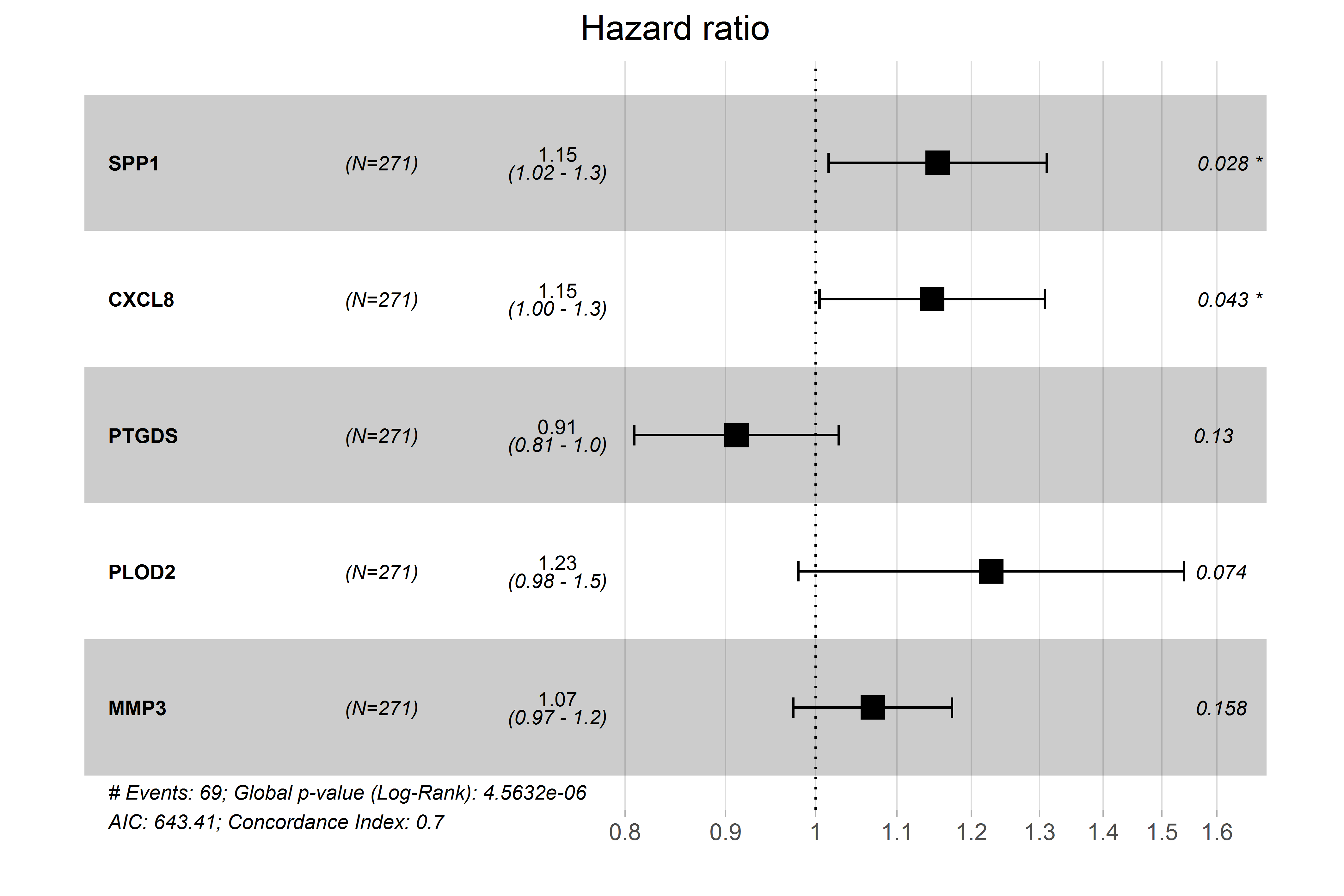

Supplement: Supplementary file 11 — Additional file 11: Figure S11. Five genes were screened by Multivariate cox regression analysis. [file 12885_2021_8212_MOESM11_ESM.tiff]

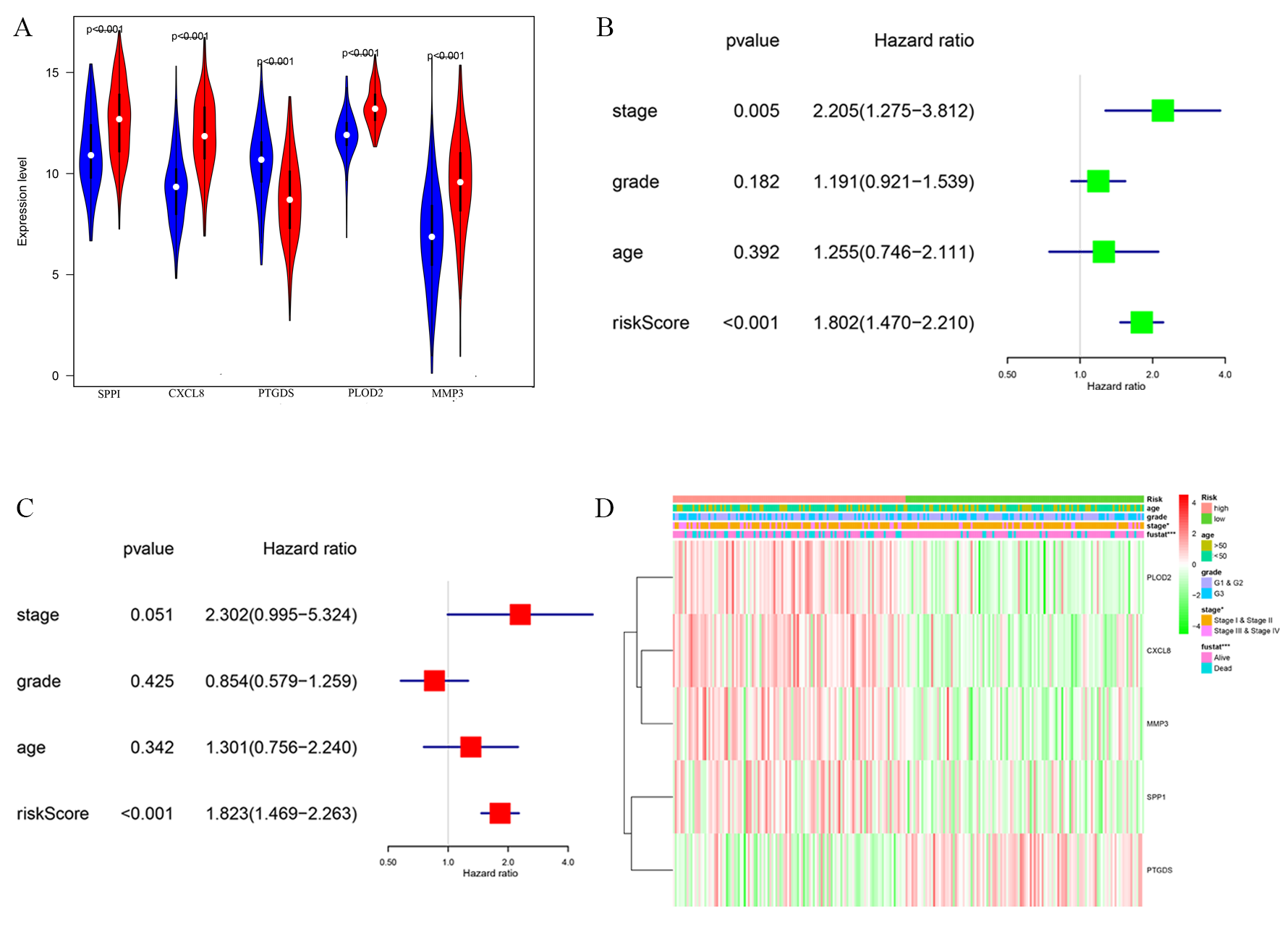

Supplement: Supplementary file 12 — Additional file 12: Figure S12. Regression analysis of the 5 genes identified. (A) The expression level of the 5 genes in low- and high-risk groups. (B) The univariate Cox proportional hazards regression. (C) The multivariate Cox proportional hazards regression. (D) The heatmap of the 5 genes in high- and low-risk patients in TCGA dataset. [file 12885_2021_8212_MOESM12_ESM.tif]
